# Round the Hospitals

**Published:** 1919-01-25

**Authors:** 


### ROUND THE HOSPITALS.

Genuine sympathy will be felt by all members of the nursing service with the King and Queen in the painful circumstances surrounding the illness and death of their youngest son, Prince John. None know better than nurses the terrible strain of anxiety entailed in the case of a child suffering from an almost incurable malady. Her Majesty's ready and tender sympathy with sick children displayed in intercourse with them by winning their confidence and in tireless efforts on their behalf reveal a heart attuned by personal experience to feel for other mothers. That the comfort Queen Mary has been the means of bringing into countless homes may be reflected back, multiplied a hundredfold, to the Royal parents in this hour of sorrow will be the earnest hope of all their devoted subjects.

Some delay has occurred in setting up the machinery for dealing with the question of salaries by the Council of the College of Nursing. It is extremely necessary that no hurried pronouncement should be made on a question of such vital importance to every member of the College. Not only are there many branches of the profession, each one of which demands patient investigation, but in each branch there are distinctions. There are small and large hospitals; some have medical schools and some have not. The differences between provincial and London, special and general institutions have to be kept in view. To ignore facts and generalise hastily would certainly inflict great injustice on individuals, and might conceivably retard the natural upward trend now taking place under the influence of demand and supply. The matter is not one which

**ROUND THE HOSPITALS**—(*continued*).

can advance rapidly at a moment when every institution is reorganising on its passage from a war to a peace footing, and while the havoc wrought by the recent epidemic is hardly yet repaired.

Nurses' salaries ought not to be considered solely in their relation to nurses and their employers. The isolation in which nurses work as regards other women is not for their good. The tacit assumption that rules made for the guidance of employers in other occupations open to women can safely be ignored when it is a question of nursing has damaged the profession, and ought to be dropped. We understand that the Council of the College has decided to invite representative women from other professions to confer with them in relation to the whole question of salaries and conditions of labour, a course which cannot fail to commend itself to those best qualified to judge. The question of salaries cannot be considered apart from that of emoluments, and in this branch of the subject the standard set in other professions is likely to be of considerable importance. It is not generally known that the College of Nursing is affiliated to the National Union of Women Workers and sends representatives to sit on four of its committees—those dealing with public health, with education, with legislation, and with child welfare.

Nothing has illustrated the vitality of the College more forcibly than the recent series of Conferences held throughout the country to consider the best way of promoting union and affiliation between general hospital training-schools and the special and small hospitals. All the local centres were invited to hold Conferences on the subject, and fourteen have done so. In each case a report has been drawn up, and these reports will be presented by the Local Representative to the Council, who will report on the entire subject and probably refer it to the Curriculum Committee for further consideration. The local centres have thus solved the initial difficulty of getting nurses in many different localities to participate in the attempt to form agreement on this puzzling question.

It is now demonstrably proved that the opponents of the College who have continued their propaganda of misstatement and falsehood for a lengthened period, without success, are almost in despair. This result is but just, and their final conspiracy by false representations to shake the confidence of the nurses in their Own College gives the nurses their opportunity to expose and silence them once for all. Like the Bolshevists, the enemy are so bent on getting their way, even at the expense of the destruction of trained nurses' best interests, that an organised attack is being made on individual nurses who have joined the College Register. This organised effort to injure the College and its members aims at securing resignations from the College by mixing up the action of the Joint War Committee with its Scholarship scheme, with which the College has no connection whatever, so as to lead nurses to believe that the College, not, as they know to be the fact, the British Red Cross Society and the Order of St. John, is granting a sum of money for the purpose of bestowing scholarships for the training of V.A.D.s and other voluntary workers in hospitals. Trained nurses, and especially those who are members of the College, should by this time be awake to the shamelessness of the false statements which the College opponents make it their business continuously to circulate. We hear, however, that some of the busier nurses have been disturbed already by fearing the College is in some way mixing itself up with the propaganda to put V.A.D.s before themselves. It seems incredible that falsehoods of this description should be considered, much less believed, by fully-trained Nurses. We hope that this plain warning of what is before them will fire the mettle of every working nurse throughout the country to unite to expose and denounce this outrage upon the nurses who have already joined the College Register. Then all College nurses may properly rely on their sisters to unite with them to bring the numbers on the College Register to at least 20,000 in the shortest possible time.

All apprehension on the part of nurses that the College, or any one else for that matter, is taking steps to secure a three years' certificate of training for V.A.D.s without going through three whole years' training at a recognised training-school is ridiculous on the face of it. Here are the facts. If V.A.D.s had the opportunity, and were to take a two years' course of training, they would never obtain one of the higher positions in any Service, and consequently they would not qualify for pensions. This position has brought into line everybody who has to do with the training of nurses in regard to this matter. Those training-schools which require three years' training before granting a certificate clearly cannot admit a V.A.D. or any other woman to training on any other conditions. The heads of training-schools feel, and one of the foremost of British matrons points out, that one of the strongest reasons—setting aside professional status—why she could not be a party to advocating a short course of training for V.A.D.s who have served during the war in military or auxiliary hospitals is that to do so would be to mislead applicants for training, and to damage their prospects by depriving them of the advantages which they had justly a right to expect. Every V.A.D., like every other woman who enters nursing as a profession, must realise that a three years' certificate of training at a recognised training-school is the only certificate, that will constitute her a fully-trained nurse, admit her to the Services, and qualify her for a pension. We hope this bogey, started by those who are ambitious to lead the nursing profession, though they continuously demonstrate that their statements cannot be trusted by any intelligent nurse, who is alive to her own interests, may henceforward be disregarded, and treated with the contempt to which it is justly entitled. The best answer of every trained nurse is to put her whole heart into making Her College the Bulwark and Defence for the Working Nurse which it will rapidly prove itself to be.

**ROUND THE HOSPITALS—(*continued*).**

We understand that a great number of probationers in the London hospitals are daughters of military officers and professional men. They were placed there by their parents, who knew they could easily get into one of the Services and rise to the highest positions therein as holders of a certificate from one of the London training-schools. A pension is assured to them in this way unless they marry; and many officers have no opportunity of making other provision for their children than this. These facts are closely connected with the warning to V.A.D.s, and with the reassurance to fully-trained nurses given in the preceding paragraphs.

A rise of salaries is reported from Bedford County Hospital, and we welcome the announcement that the matron is not this time left out. The curious mystery maintained about matrons' salaries is not justifiable on any theory. It was a purely mid-Victorian notion that matrons were supposed to work solely for the love of their kind, and it has too long been suffered to prevail. A minute salary, amounting in effect to a mere honorarium, has often been all that the governors have thought fit to bestow on women whose organising powers would have brought them affluence in any other career. We note that the matron at the Bedford County Hospital is now to receive £160 a year. The assistant matron receives £65, rising by £5 increment to £80. The children's and theatre sisters receive £46 to £54 (these are underpayments and should be increased.—Ed. The Hospital); sisters of men's and women's wards, £52 to £60; the night sister, £55 to £63; staff nurses, £38 to £44; probationers, £14, £20, £26. All salaries to date under the new scale from January 1 last.

Nursing committees are still apt to demur at granting their nurses permission to attend unmarried mothers, and the Local Government Board's decision that midwives should attend all cases of confinement when desired has proved exceedingly unpopular in Billericay, where local nursing association committees have resigned in consequence. The question is more complicated than it seems to be at first sight. The unmarried mother is usually in need of far more than the nursing association can provide. She can be more satisfactorily dealt with as an in-patient, and if the accommodation offered her by the Poor Law is such that she rejects it with dismay the discredit should rest on the ill-managed workhouse. As things are, the nursing associations with but few exceptions shoulder their responsibilities towards the unmarried mother with great discretion and liberality. But the Poor-Law system should be condemned and executed, if for nothing else, for its unworthy callousness to the young women whom it debases in the very act of relieving.

Shall mothers be paid for bringing up their children? This is the proposition with which the nation will soon be asked to deal, and on examination many of the obvious objections disappear. An immense ramification of institutions, doctors, and nurses has to be established and kept going because mothers fail in their elementary duties to their offspring. They fail for two reasons. The first is poverty, which obliges them to leave their children in order to supplement the family income. Were they to receive an allowance from the State this root cause of neglected childhood would cease to exist. But they also fail from ignorance and incapacity. How could State pensions remedy this? The State Children's Association has been accumulating information on this point, and has brought over a very large number of district councils, health societies, and kindred associations to its views. No fewer than 200 Parliamentary candidates have expressed their approval of the scheme as formulated. In short, the propaganda in favour of mothers' pensions has now reached the stage when action may be looked for.

The death of Miss Mary Clifford at Bristol, aged seventy-six, and the touching memoir of her furnished by the *Times* of the 11th inst., bring back to mind the days when women felt life could hardly be endured after their names had been exposed in the public streets on posters four feet long. Miss Clifford dared to brave the ordeal and was one of the first women guardians to be elected in 1882, an office she held for twenty-five years. Her main principle in philanthropic efforts was to uphold the value of personal relations with the poor as individuals. She was one of the pioneers in promoting the boarding-out system, and did splendid uphill work in promoting the cause of feeble-minded women and girls. She took an influential part, with her brother Mr. Edward Clifford, in starting and developing the Church Army, and was a humorous, lucid, and telling speaker. Her lofty spiritual outlook, added to a singularly gracious charm of manner, made her a powerful social influence. To many of our readers she was personally known and a valued friend and counsellor.

It seems but a short while ago, but in reality it must be close on twenty years, since the Middlesex Hospital was rejoicing in a new Nurses' Home, which was then esteemed a model of its kind. Long ago, however, the growth of the hospital, which has added ever new departments, outstripped the accommodation for its nurses. And Princess Alice, on behalf of the governors, is now making an appeal for the sum of £50,000 wherewith to build and equip a new Nurses' Home. Already £3,000 has been received, and, considering the urgent need for developing the resources of the major nurse training-schools, there can hardly be a doubt that the sum needed will speedily be forthcoming. Nurses' homes are no longer mere hostels for housing and feeding the persons engaged in nursing duties. They are centres of collegiate life. It has become only too clear that the character and outlook of the probationers are profoundly modified by their environment outside the wards. The effect of continued association with morbid processes, with disease, pain and death, needs counteracting if women are not to be warped in their passage to the

**ROUND THE HOSPITALS—(*continued*).**

status of a trained nurse. Hence the expense of the modern nurses' home.

An excellent scheme for utilising a war depôt in reconstruction work has been framed by the authorities of the Belgravia War Hospital Supply Depôt, 4 Grosvenor Crescent, S.W. 1. This depôt, which has four branches in London in addition to fifty-two country branches, has sent nearly five million articles for the use of the wounded since it was started in October 1915. It now proposes, with the sanction of the D.G.V.O., to undertake work during the next two months for the refugee population of the Allies. It has selected as the centre of its operations the ruined district of Montdidier. In this terribly devastated region a French village will be chosen (probably more than one), and the depôt will undertake the weighty task of finding clothing for the destitute refugees, of helping them back to their homes, and surrounding them once more with some of the comforts of civilisation. As vast numbers of these underfed and cruelly impoverished people, especially the young and the aged, are in great need of medical and nursing care, dispensaries will be established in every village. The Belgravia War Depôt also proposes to send aid to the Serbian Relief Funds by equipping civilian hospital centres in Serbia. Gifts of blankets and partly worn garments which are in good condition are invited, and it is hoped that auxiliary hospitals which are closing will contribute articles towards this good work.

THE Victory Ball at the Albert Hall has brought in the sum of £16,520 18s. 7d. in aid of the Nation's Fund for Nurses. Dame May Whitty, O.B.E., and Viscountess Cowdray are to be warmly congratulated for the able organisation which brought about such a fine result with but a fortnight's preparation. The next ball is to be in aid of the King's Fund for Sailors. In view of the fuss and worry of a bazaar and the increased reluctance of the public to purchase things they do not need, the process of dancing the coffers full has much to recommend it.

SISTER MCCREATH, who has been in charge of the Newtown (Cumberland) War Hospital since it was opened in 1917, has received a handsome silver tea-service from her grateful soldier patients as a token of their admiration and respect. The hospital has gained a high reputation in the neighbourhood, and it has been proved once more that the soldier patient who is treated with consideration invariably proves a very good fellow. We trust Sister McCreath will have an opportunity elsewhere, when the hospital is closed, of exercising her undoubted gifts of organisation.

THE attempt of the Royal British Nurses Association to form a league of nurses engaged in Public Health work is a sign of the increased importance now attached to this branch of work. But we fear the difficulties of centralisation in this direction will militate against the attainment of large numbers. Public Health nurses are scattered all over the Kingdom and can rarely find the opportunity of meeting in council. Their interests are substantially the same as those of other members of the profession, and it is particularly important that they should be influentially represented on the Council and Local Centres of the College, for they could profoundly modify the curriculum of training-schools as it relates to women aspiring to municipal posts. There is room for combination in branches of nursing, but the really vital matter at the moment is to combine in order to get a Central Nursing Authority set up without delay.

THE movement in favour of establishing Cottage Hospitals as War Memorials becomes stronger every day, partly because people are justly suspicious of local ability to evolve memorials of artistic beauty, and partly because hospital work is interesting an unusually large public at the present time, since many prominent local people have aided in it as V.A.D. Commandants and members. It is very important, in our judgment, that these schemes should be formed in co-operation with the Health authorities whoever they may prove to be, and should not be isolated from Infant Welfare and Child Clinic undertakings. In many small towns where a tiny memorial hospital might languish after the first enthusiasm of founding it had subsided, it could become a live and valuable establishment were it linked with a Welfare Centre and provided with properly equipped children's and maternity wards. The difficulty of staffing would then disappear, for the combined institution would be justified in appointing a thoroughly competent matron, a matter on which the entire success of Cottage Hospitals has been proved to hinge.

DR. AMAND ROUTH, consulting obstetric physician at Charing Cross Hospital, gave startling evidence before the National Birth-Rate Commission on the 20th inst. He showed by statistics that apart from the effect of sterility and the artificial restriction of child-bearing, ante-natal death and disease were the most serious causes of a low birth-rate, for if the unborn child did not perish during gestation it was born so debilitated that it failed to survive its birth or only lived a few hours or days. Reckoning the number of still births in 1914, *i.e.*, deaths of children within the last twelve weeks of gestation, now notifiable, estimating that four times as many died within the twenty-eight earlier weeks, and reckoning in addition the number of infants who died before the end of their first year of life, Dr. Routh demonstrated that the proportion of deaths during this period from conception to the end of the first year amounted to 250 in 1,000 or one in four. These facts cannot be too widely known. While stimulating scientific research into the hitherto unidentified toxins which war against infant life, they indicate as forcibly the need for a vast accession of strength to the midwifery and public health services of the whole country.

